# Constructive Charity

**Published:** 1888-09-22

**Authors:** 


### The Hospital

A Weekly Institutional Journal of

Science, Medicine, Nursing, and Philanthropy.

*For Hospitals, Asylums, Medical Practitioners, Students, Nurses, Families, Parishes, Congregations, and the Charitable Public.*

Vol. IV. No. 104.] [September 22, 1888.

### Constructive Charity.

The Charity Organisation Society is an example of an association which, whilst performing functions highly necessary and useful, hardly expresses the nature of its work by its designation. It is not so much an "organising" society as a critical and inquisitorial organisation. All men who take the trouble to look beneath the surface of human affairs know the necessity which exists for doubt, inquiry, and criticism. He who proposes to accomplish anything effectual for his fellow-men must look before he leaps—must see and understand before he can know and do. But seeing and knowing are comparatively easy efforts; it is when we approach the period of construction, of actual putting forth of the hand to effective work, that difficulties begin. It is not the man of criticism, but the man of results on whom his contemporaries lay odds.

It is probable that the various charitable institutions of this country spend not less than eight millions sterling every year. Their annual income will reach that sum at least; it may be a great deal more. But, though the amount of money spent annually in charity is so enormous, there is no generally recognised central organisation of an authoritative or even of an advisory character. The charitable world is neither an empire, a kingdom, nor even a well-ordered parish. It can only be described as a "Chaos of Benevolence." It resembles the German Confederation in pre-Bismarckian days; and is as feeble and ineffectual in attack and defence as that multitudinous agglomeration of State incapabilities proved itself to be. For want of a head, co-ordination, concerted action, is impossible, and the huge creature flounders about like one of the larger amphibians surprised at a distance from the water.

But organisation is imperative if charity as an effective force is to survive. The sources of supply must flow into recognised and definite channels, or they cannot ultimately be distributed with fruitful results. If they are not guided into definite and right channels, they spread over the country as devastating floods rather than as healthful and life-giving streams. When the population was smaller, when large towns were fewer and more limited in extent, each town could provide its own hospital and other charities, and could keep them well in hand by effectual supervision and criticism. But in these times, and in this country, towns are grown to states, and each state has not one hospital, but many; whilst the metropolis, the largest state of all, numbers its separate hospitals by the hundred. The task of supervision by town authority is felt to be hopeless; and criticism is perceived to be of so desultory and ineffectual a character that many who are most competent cease to attempt it.

An informing brain is wanted, a brain and spinal cord with an afferent and efferent nervous system. The brain should be so clear, so capable, so practical, and so persuasive that every separate charitable institution, however small, would gladly run to avail itself of the central light and guidance. It must be a brain, not merely critical, but constructive. It should be able to show, from a large knowledge and experience, what had been done of the foolish, wasteful, and ineffectual kind in the past, and also what had been effected which was wise, economical, and successful. From its resources it should be able to check at once and certainly all the reckless and wicked waste which may now be witnessed in every large town, and also to lead, with the authority of convincing reason, all the foolish followers of the army of charity into the ways of discipline and successful campaigning. It should not, and must not, aim at despotic centralisation. Its object should be to seek and attract the light from every quarter; and from its own storehouse to irradiate all the darkest and remotest corners of the entire charitable world. There would be no necessity for any such head to claim rule and government. The proper rule that would belong to it would be the authority of superior knowledge, proved discretion, and the power of wise guidance. To such authority all reasonable men are always ready to yield assent and obedience.

The practical man says: All this is most true, but where is your central brain, with its comprehensive wisdom and constructive power, to come from? Does the practical man believe in the doctrine of development? Is he an evolutionist of the Darwinian or of the Spencerian type? If he is, he cannot surely despair. Given an organism and an environment, it seems certain that the organism will sooner or later adapt itself to its environment. It will, at any rate, either do that or perish. Now hospitals, and many other charitable institutions, are survivals from a period considerably remote. They are not of yesterday. Hitherto they have proved themselves capable of survival, and not merely of survival, but of progressive development in a variety of directions. Is it to be believed that they have reached their ultimatum, and will now gradually become extinct? We, at any rate, do not believe it. So far as our judgment serves us, there is before them a period of rapid development on sound and permanent lines.

But the central brain is now demanded in loud and imperative tones. Where is it? The nucleus of it is at least evident, and it has originated within the hospitals themselves. The Hospitals Association of London is the distinct answer of the organism to the pressure of its environment. We do but here give utterance to thoughts and purposes which have exercised the minds of all earnest hospital managers for many years past. They feel as keenly as we do that the demands of a critical public are founded in reason; and they are resolved, so far as lies in their power, to manage the institutions committed to their charge as wisely, as economically, and as efficiently as it is possible for them to do. The Hospitals Association now consists of representatives of a hundred and fifty charitable institutions. It desires to represent every hospital and charitable institution in the United Kingdom. It already possesses a vast amount of collected, digested, and stored information concerning medical charities in every part of the world. It has won the confidence of hospitals not only by its zeal, but by its patience and its discretion. In its president, Dr. Bristowe, it has a head whom the great world admires and trusts, and whom the smaller world, which knows him best, esteems and loves. Under his guidance the Association has rapidly advanced in the confidence of hospitals and the public. The session which closed in May was the most diligent, the most energetic, and the most useful it has known.

The Hospitals Association is not merely or mainly critical; it aims at being constructive. The difficulties of hospitals, the doubts of subscribers, the necessities of patients—these are the subjects which engage its attention, and on which it strives to throw the light of its garnered knowledge and the wisdom of its wide and growing experience. There is before its members a possibility of usefulness second to that of no voluntary institution. In seeking to form charitable opinion and to give practical guidance in all hospital affairs it aims at a career worthy of the wisest minds and the best hearts of the time.

